# Carbapenem-resistant *Enterobacter cloacae* complex in a tertiary Hospital in Northeast China, 2010–2019

**DOI:** 10.1186/s12879-021-06250-0

**Published:** 2021-06-26

**Authors:** Jingjing Chen, Sufei Tian, Hua Nian, Ruixuan Wang, Fushun Li, Ning Jiang, Yunzhuo Chu

**Affiliations:** 1 Department of Laboratory Medicine, The First Affiliated Hospital of China Medical University, 155 North Nanjing Street, Heping District, Shenyang, 110001 Liaoning China; 2 National Clinical Research Center for Laboratory Medicine, The First Affiliated Hospital of China Medical University, Shenyang, 110001 China; 3 Labortory Medicine Innovation Unit, Chinese Academy of Medical Sciences, Shenyang, China

**Keywords:** *Enterobacter cloacae* complex, Carbapenem-resistant, Molecular analyses, ST93, NDM

## Abstract

**Background:**

Carbapenem-resistant *Enterobacter cloacae* complex (CREC) is a new emerging threat to global public health. The objective of the study was to investigate the clinical characteristics and molecular epidemiology of CREC infections in the medical center of northeast China.

**Methods:**

Twenty-nine patients were infected/colonized with CREC during a ten-year period (2010–2019) by WHONET analysis. Antibiotic susceptibilities were tested with VITEK 2 and micro broth dilution method (for polymyxin B and tigecycline). Carbapenemase encoding genes, β-lactamase genes, and seven housekeeping genes for MLST were amplified and sequenced for 18 cryopreserved CREC isolates. Maximum likelihood phylogenetic tree was built with the concentrated sequences to show the relatedness between the 18 isolates.

**Results:**

There was a rapid increase in CREC detection rate during the ten-year period, reaching 8.11% in 2018 and 6.48% in 2019. The resistance rate of CREC isolates to imipenem and meropenem were 100.0 and 77.8%, however, they showed high sensitivity to tigecycline, polymyxin B and amikacin. The 30-day crude mortality of CREC infection was 17.4%, indicating that it may be a low-virulence bacterium. Furthermore, molecular epidemiology revealed that ST93 was the predominant sequence type followed by ST171 and ST145, with NDM-1 and NDM-5 as the main carbapenemase-encoding genes. Moreover, *E. hormaechei* subsp. *steigerwaltii* and *E. hormaechei* subsp. *oharae* were the main species, which showed different resistance patterns.

**Conclusion:**

Rising detection rate of CREC was observed in a tertiary hospital, which showed heterogeneity in drug resistance patterns, resistance genes, and MLST types. Effective infection prevention and control measures should be taken to reduce the spread of CREC.

**Supplementary Information:**

The online version contains supplementary material available at 10.1186/s12879-021-06250-0.

## Background

*Enterobacter cloacae* complex (ECC) which comprises the following species, *Enterobacter cloacae*, *Enterobacter hormaechei*, *Enterobacter asburiae*, *Enterobacter kobei*, *Enterobacter ludwigii*, *Enterobacter nimipressuralis, Enterobacter mori*, etc., is an important *Enterobacteriaceae* widely encountered in the environment [1]. As an opportunistic pathogen, it has ranked as the top three *Enterobacteriaceae* in hospital-associated infections these years. ECC are found to be involved in multiple infections, such as bacteremia, respiratory tract infections, wound infections, urinary tract infections, nosocomial infections, etc. [2].

To date, carbapenem-resistant *E. cloacae* complex (CREC) has become as the third most common carbapenem-resistant Enterobacteriaceae (CRE) in China [3]. According to the surveillance of China Antimicrobial Surveillance network (CHINET), carbapenem resistance rates among ECC were < 1.0% in 2007. Surprisingly, it rapidly increased to about 10% in 2019. Carbapenems are regarded as a last choice for treating severe gram-negative bacterial infections. Although the consequences of CREC infections remain largely unknown, infections caused by CRE can lead to high mortality, long hospitalization and high hospitalization cost. Therefore, CREC may become a new emerging threat to public health [4].

Genes encoding carbapenemases (KPC, NDM, VIM, IMP, and OXA-48), which are usually present on the plasmids, are the main mechanism of carbapenem-resistance in CREC. Besides, overexpression of β-lactamases encoded by TEM, CTX-M, SHV, etc., membrane-associated mechanisms, such as porin defects (Omps, porins, and outer membrane permeability), and efflux pumps may also participate in carbapenem resistance [1]. Global surveillance showed diversification of sequence types and resistance genes in ECC. Besides, regional distribution of CREC is observed, with KPC predominant in North America, OXA-48 and VIM predominant in Europe, and NDM predominant in China [5].

Due to the unclear clinical characteristics and notable diversity of CREC, this study was therefore conducted to investigate the clinical characteristics and molecular epidemiology of CREC infection/colonization in the First Hospital of China Medical University, which is the medical center in northeast China. The present study will contribute to understanding this emerging carbapenem-resistant pathogen, which are fundamental for further treatment, effective infection prevention and control.

## Materials and methods

### Bacterial strains

This study was performed in the First Affiliated Hospital of China Medical University, a tertiary teaching hospital with 2249 beds and also the medical center of northeast China. CREC was defined as *E. cloacae* complex strains resistant to imipenem or meropenem. Twenty-nine patients were infected/colonized with CREC from January 2010 to December 2019 through WHONET analysis. Determination of infection and colonization of the patients was performed by two clinicians. Among these, 18 isolates (one isolate per patient) were cryopreserved and further experiments were carried out. 16 s rRNA sequencing was performed to confirm the species, and *hsp60* typing was applied to discriminate the genetic clusters [6].

This is a retrospective study which was approved by the Medical Ethics Committees of the First Affiliated Hospital of China Medical University. The Medical Ethics Committees of the First Hospital of China Medical University waived the need of informed consent.

### Species identification and antimicrobial susceptibility testing

The VITEK 2 system and the MALDI TOF MS (bioMérieux, France) were applied for isolate identification, and the VITEK 2 GN09 was used to test the antimicrobial susceptibilities of all isolates. Carbapenem resistance was verified by Etest or K-B diffusion method. Minimal inhibitory concentrations (MICs) of polymyxin B and tigecycline were determined by broth microdilution method for the 18 isolates. The resistance results of tigecycline were interpreted following the European Committee on Antimicrobial Susceptibility Testing (EUCAST) guidelines. Susceptibilities of the other drugs were determined by the criteria of Clinical and Laboratory Standards Institute (CLSI). All methods were carried out in accordance with relevant guidelines and regulations.

### Phenotypic screening for carbapenemases and sequencing of antimicrobial resistance genes

The phenotypic detection of carbapenemases production was achieved by RAPIDEC CARBA NP (bioMérieux, France). Carbapenemase genes (KPC, NDM, IMP, VIM, and OXA48-like), β-lactamase genes (TEM, CTX-M, and SHV) and MCR-1 were amplified by polymerase chain reaction (PCR) according to previous methods [7], then positive PCR products were subjected to commercial Sanger sequencing services (Beijing Genomics institution Co., Ltd., China). Sequences were analyzed by nucleotide homology comparison against GenBank database by BLAST.

### Multilocus sequence typing (MLST)

The *E. cloacae* MLST was performed as described previously (https://pubmlst.org/ecloacae/) by sequencing seven housekeeping genes: dnaA, fusA, gyrB, leuS, pyrG, rplB, and rpoB [8]. Briefly, the genes were amplified and then DNA sequencing was performed for positive ones. Sequence types (ST) were assigned by uploading the sequences to the online *Enterobacter cloacae* typing database.

### Phylogenetic analysis

The sequences were assembled with Contig software, edited with BioEdit and aligned with Clustal W tool present in BioEdit software. After combining the seven MLST genes and 16S rRNA gene together, maximum likelihood phylogenetic tree was constructed with MEGA 5.1 software to display the relatedness between the 18 isolates. Bootstrap analyses with 1000 replicates were applied.

### Statistical analyses

Statistical analyses were performed with WHONET software 5.6 and SPSS 20.0 software. For all statistical analyses, *p* value < 0.05 was considered statistically significant.

## Results

### Basic characteristics of the CREC isolates

A total of 29 consecutive nonduplicate CREC isolates were identified during 2010–2019 (Table 1), which originated from different anatomical sites: urine (*n* = 8, 27.6%), blood (*n* = 6, 20.7%), drainage (*n* = 5, 17.2%), sputum (*n* = 5, 17.2%), puncture fluid (*n* = 1, 3.4%), catheter (*n* = 1, 3.4%), tissue (*n* = 1, 3.4%), secretion (*n* = 1, 3.4%), and semen (*n* = 1, 3.4%). The majority of patients were in the intensive care unit (*n* = 9, 31.0%), followed by hepatobiliary surgical ward (*n* = 3, 10.3%), urinary surgery ward (*n* = 3, 10.3%), respiratory ward (*n* = 3, 10.3%), cardiac surgery ward (*n* = 2, 6.9%), surgical clinic (*n* = 2, 6.9%), orthopedic ward (*n* = 1, 3.4%), hematology ward (*n* = 1, 3.4%), neurosurgery ward (*n* = 1, 3.4%), neonatal ward (*n* = 1, 3.4%), infection ward (*n* = 1, 3.4%), otolaryngology clinic (*n* = 1, 3.4%), and emergency center (*n* = 1, 3.4%).

**Table 1:** Basic characteristics of included patients infected/colonized with CREC

Isolation ID	Specimen	Infection/colinization	Patient age^a^	Sex	Isolation date^b^	Department	Outcome^c^
CMU1	blood	Infection	51	F	2012/4/18	Hematology ward	Survive
CMU2	secretion	Infection	60	M	2014/9/17	Otolaryngology clinic	Survive
CMU3	urine	Colonization	77	F	2015/4/27	Intensive care unit	Survive
CMU4	urine	Colonization	57	M	2015/5/9	Urinary surgery ward	Survive
CMU5	tissue	Infection	29	M	2015/6/17	Orthopedic ward	Survive
CMU6	urine	Infection	60	M	2016/8/18	Surgical clinic	Survive
CMU7	drainage	Infection	52	M	2016/12/21	Intensive care unit	Die
CMU8	urine	Infection	86	M	2017/1/11	Intensive care unit	Survive
CMU9	urine	Colonization	11	M	2017/4/29	Infection ward	Survive
CMU10	sputum	Infection	81	F	2017/7/20	Intensive care unit	Survive
CMU11	drainage	Infection	66	M	2017/8/4	Hepatobiliary surgery	Survive
CMU12	drainage	Infection	54	M	2017/9/12	Hepatobiliary surgery	Survive
CMU13	puncture fluid	Infection	66	M	2017/11/7	Emergency center	Survive
CMU14	urine	Infection	72	M	2018/5/11	Respiratory ward	Survive
CMU15	urine	Colonization	61	M	2018/5/29	Urinary surgery ward	Survive
CMU16	semen	Infection	54	M	2018/6/13	Surgical clinic	Survive
CMU17	blood	Infection	8 days	F	2018/6/18	Neonatal ward	Survive
CMU18	blood	Infection	54	M	2018/7/12	Respiratory ward	Survive
CMU19	blood	Infection	68	F	2018/7/19	Neurosurgery ward	Survive
CMU20	sputum	Colonization	82	M	2018/10/18	Respiratory ward	Survive
CMU21	blood	Infection	16	M	2018/11/22	Intensive care unit	Survive
CMU22	sputum	Infection	60	M	2018/12/19	Intensive care unit	Die
CMU23	sputum	Infection	77	M	2019/1/3	Hepatobiliary surgery	Survive
CMU24	catheter	Infection	52	M	2019/3/4	Cardiac surgery ward	Die
CMU25	sputum	Colonization	60	M	2019/3/22	Cardiac surgery ward	Survive
CMU26	blood	Infection	21	M	2019/4/11	Intensive care unit	Survive
CMU27	drainage	Infection	60	F	2019/5/4	Intensive care unit	Die
CMU28	drainage	Infection	33	M	2019/7/12	Intensive care unit	Survive
CMU29	urine	Infection	76	M	2019/11/17	Urinary surgery ward	Survive

^a^ years

^b^ year/month/day

^c^ survive or die within 30 days

**Fig. 1:**
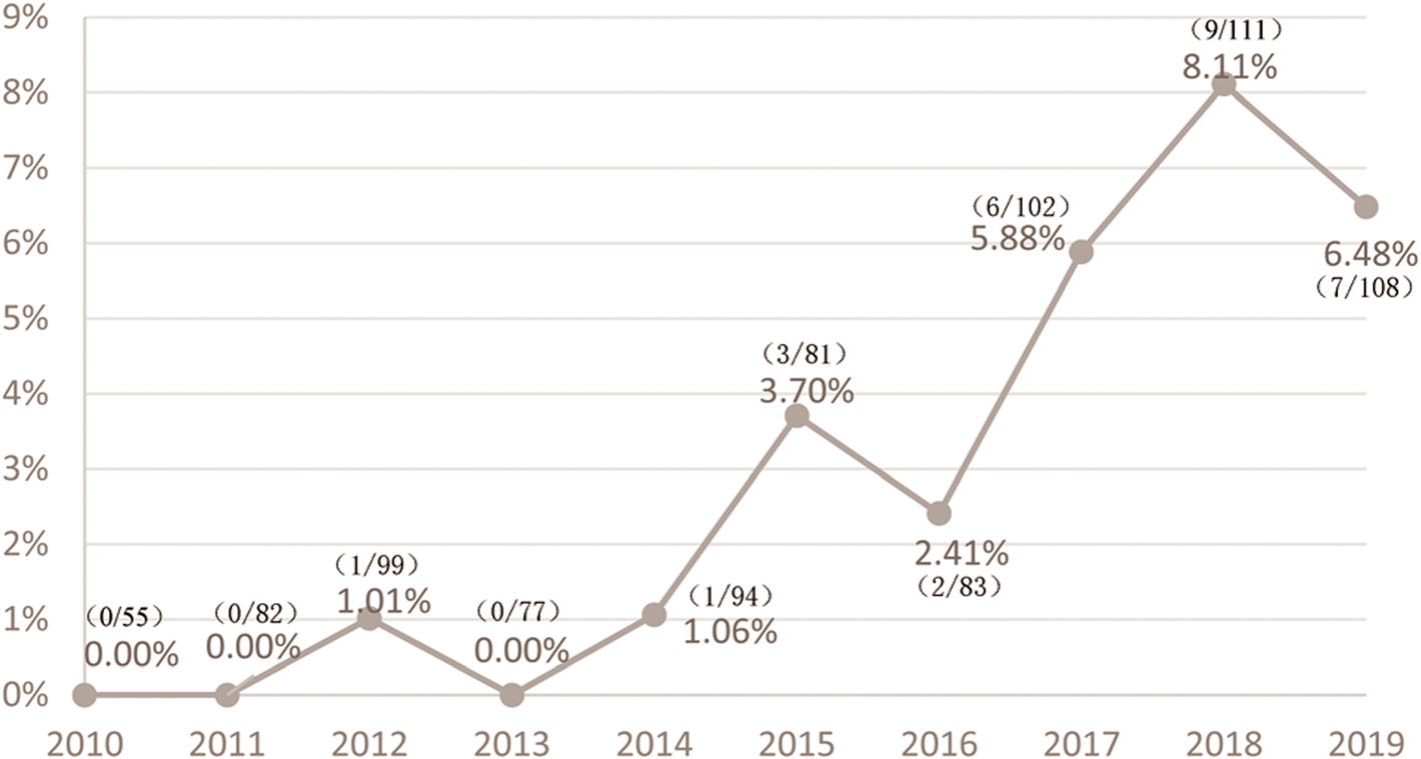
CREC detection rate during 2010–2019

The first isolate of CREC dates back to 2012 in the hematology ward. During the 10-year period, the CREC detection rate increased notably from 0.00% in 2010 to 6.48% in 2019, with a peak of 8.11% in 2018 (Fig. 1). This rapid increase deserves further molecular epidemiology research.

### Clinical outcomes of CREC infections

The overall 30-day crude mortality of CREC infection was 17.4% (4/23). The specimen types of the four patients were as follows: drainage (*n* = 2), catheter (*n* = 1), sputum (*n* = 1). None of the patients with bloodstream infections died. Furthermore, all four patients were accompanied with other infections: two patients with serious abdominal infection, one patient with cytomegalovirus pneumonia, and one patient with *Acinetobacter baumannii* bloodstream infection. This indicated that CREC may be not the main reason of death.

### Antimicrobial susceptibility testing

The antimicrobial susceptibility testing was summarized in Table 2, which showed that 100.0% (18/18) isolates and 77.8% (14/18) isolates were resistant to imipenem and meropenem respectively. Among them, nine isolates showed extreme resistance (MIC ≥16) to both drugs. In addition, these isolates showed high resistance to ceftriaxone (94.4%), ceftazidime (94.4%), piperacillin/tazobactam (77.8%), cefepime (72.2%), ciprofloxacin (66.7%), and levofloxacin (61.1%). Moreover, 55.6, 55.6, 44.4 and 44.4% strains were resistant to gentamicin, sulfamethoxazole-trimethoprim, nitrofurantoin and tobramycin respectively. In contrast, 100, 100 and 77.8% of the isolates were susceptible to tigecycline, polymyxin B and amikacin.

**Table 2:**
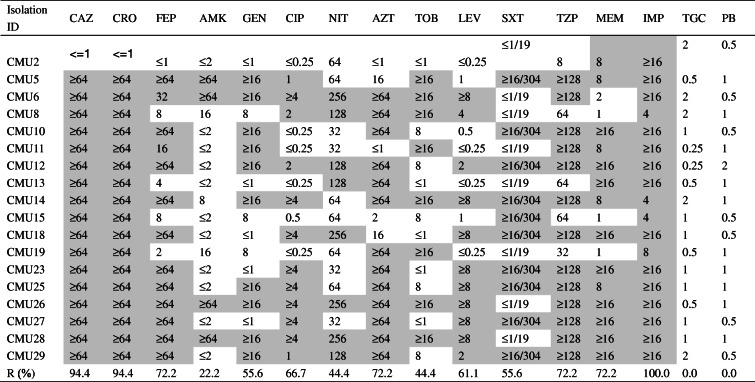
Antimicrobial susceptibility results showing the MICs of 18 CREC isolates

Grey-shaded cells were interpreted as resistant

*Abbreviations*: *CAZ* ceftazidime, *CRO* ceftriaxone, *FEP* cefepime, *AMK* amikacin, *GEN* gentamicin, *CIP* ciprofloxacin, *NIT* nitrofurantoin, *AZT* aztreonam, *TOB* tobramycin, *LEV* levofloxacin, *SXT* sulfamethoxazole-trimethoprim, *TZP* piperacillin/tazobactam, *MEM* meropenem, *IMP* imipenem, *TGC* tigecycline, *PB* polymyxin B

### Resistance genes

Carbapenemase producers were detected in 94.4% (17/18) of the isolates (Table 3), and all the carbapenemase-producing isolates harbored carbapenemase-encoding genes. Among them, four types of carbapenemases were detected in these isolates: blaNDM-1 (*n* = 9, 50.0%), blaNDM-5 (*n* = 7, 38.9%), blaIMP-4 (*n* = 2, 11.1%), and blaKPC-2 (*n* = 1, 5.6%). Of note, co-occurrence of blaNDM-1 and blaIMP-4 was identified in two isolates (CMU10 and CMU29). For the β-lactamase genes, eight isolates had TEM-1, three isolates had CTX-M-15, three isolates had CTX-M-3, two isolates had SHV-12, and one isolate had CTX-M-14.

**Table 3:** Carbapenemases production, resistance genes and sequence types of 18 CREC isolates

Isolation ID	*hsp60* typing	Craba NP	Carbapenemase-encoding genes	β-lactamase genes	MCR-1	ST type
CMU2	*E. hormaechei*^c^	+	NDM-5		–	ST250
CMU5	*E. kobei*	+	NDM-5	TEM-1, CTX-M-3	–	ST145
CMU6	*E. hormaechei*^a^	+	KPC-2	TEM-1, CTX-M-3	–	ST93
CMU8	*E. hormaechei*^c^	+	NDM-1	SHV-12	–	ST66
CMU10	*E. hormaechei*^a^	+	NDM-1, IMP-4	TEM-1	–	ST93
CMU11	*E. hormaechei*^a^	+	NDM-1	TEM-1	–	ST93
CMU12	*E. hormaechei*^a^	+	NDM-1		–	ST93
CMU13	*E. ludwigii*	–			–	ST13
CMU14	*E. hormaechei*^c^	+	NDM-5	CTX-M-15	–	ST171
CMU15	*E. kobei*	+	NDM-1		–	ST145
CMU18	*E. hormaechei*^c^	+	NDM-1	CTX-M-14	–	ST114
CMU19	*E. hormaechei*^b^	+	NDM-1	SHV-12	–	ST528
CMU23	*E. hormaechei*^c^	+	NDM-5	CTX-M-15	–	ST171
CMU25	*E. hormaechei*^a^	+	NDM-1	TEM-1, CTX-M-3	–	ST1120
CMU26	*E. hormaechei*^a^	+	NDM-5	TEM-1	–	ST93
CMU27	*E. hormaechei*^c^	+	NDM-5	CTX-M-15	–	ST171
CMU28	*E. asburiae*^a^	+	NDM-5	TEM-1	–	ST93
CMU29	*E. hormaechei*^a^	+	NDM-1, IMP-4	TEM-1	–	ST93

^a^*E. hormaechei* subsp. *steigerwaltii*

^b^*E. hormaechei* subsp. *hormaechei*

^c^*E. hormaechei* subsp. *oharae*

### MLST analysis

It revealed 11 sequence types among the 18 CREC isolates, with ST93 as the predominant epidemic type (*n* = 6, 33.3%), followed by ST171 (*n* = 3, 16.7%) and ST145 (*n* = 2, 11.1%). The other types contained one isolate for each: ST13, ST66, ST114, ST528, ST1120 (*n* = 1, 5.6%).

### Clonal relatedness analysis

**Fig. 2:**
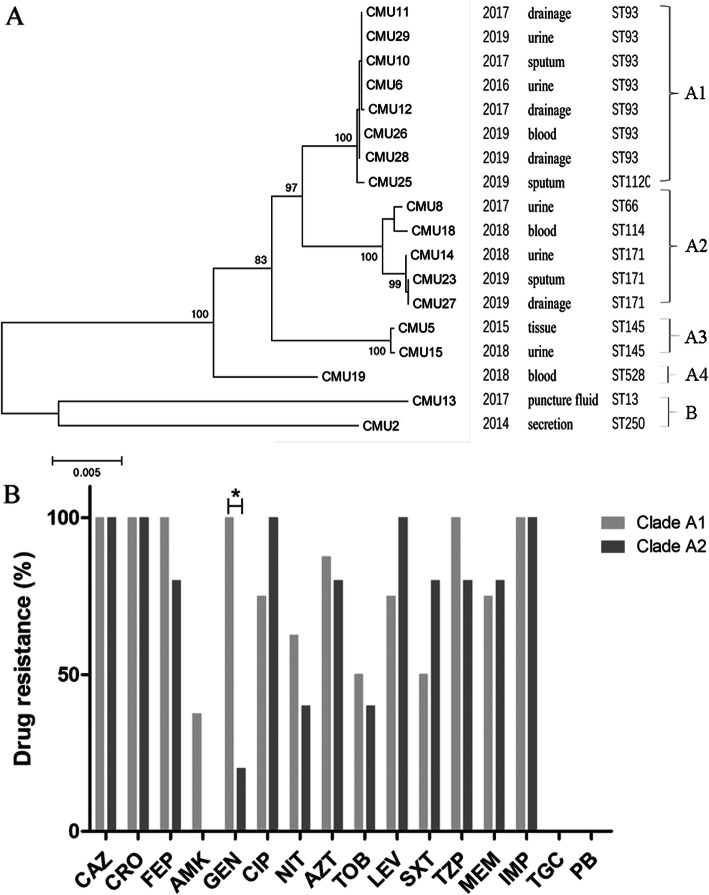
Clonal relatedness analysis of 18 CREC isolates. Maximum likelihood tree constructed with the concatenated sequences (**A**); Comparison of antimicrobial resistance rates of *E. hormaechei* subsp. *steigerwaltii* (calde A1) and *E. hormaechei* subsp. *oharae* (clade A2) (**B**)

To analyze the phylogenetic relationships between these 18 isolates, a maximum likelihood tree (Fig. 2A) was constructed with the concatenated sequences (6090 bp) of the seven loci MLST genes and 16 s rRNA, which formed two separate clades. Clade A was sub-divided into four subclades, clade A1, A2, A3, A4. Clade A1 were *E. hormaechei* subsp. *steigerwaltii* typed as ST93 and ST 1120; Clade A2 were *E. hormaechei* subsp. *oharae* typed as ST66, ST114, and ST171; Clade A3 were *E. kobei* of ST145, Clade A4 were *E. hormaechei* subsp. *hormaechei* of ST528. Whereas clade B had two sequence types and were identified as *E. ludwigii* and *E. asburiae* respectively. In short, phylogenetic analysis of 18 CREC isolates showed genetic diversity with *E. hormaechei* as the predominant species.

Furthermore, we compared the antimicrobial resistance patterns of *E. hormaechei* subsp. *steigerwaltii* (clade A1) and *E. hormaechei* subsp. *oharae* (clade A2). As shown in Fig. 2B, clade A1 were characterized with higher gentamicin resistance rate relative to clade A2 (*p* < 0.05). However, due to the small sample size, further confirmation was needed.

## Discussion

Wide spread of CREC poses a great threat to public health. Therefore, it is urgent to characterize the clinical molecular epidemiology of CREC infection in the medical center of northeast China. Results revealed that there was a rapid increase in CREC detection rate during 2010–2019, which showed high sensitivity to tigecycline, polymyxin B and amikacin through antimicrobial susceptibility test. The overall 30-day crude mortality of CREC infection was 17.4%, indicating that CREC may be a low-virulence pathogen. Besides, molecular epidemiology indicated that ST93 was the predominant sequence type followed by ST171 and ST145, with NDM-1 and NDM-5 as the main carbapenemase-encoding genes.

Since the first identification of CREC in our hospital in 2012, a rapid increase in the CREC detection rate was observed thereafter, reaching 8.11% in 2018 and 6.48% in 2019, indicating that CREC has become an escalating threat of nosocomial infection. This is consistent with previous surveillance of CHINET and the US Veterans Health Administration, which also reported an increase in the resistance rate of carbapenems in ECC [9, 10]. Reasons explaining for the increase may be as follows: (1) Extensive use of broad-spectrum antibiotics (especially third or fourth generation cephalosporins and carbapenems), invasive devices (mechanical ventilation, central venous catheter, parenteral nutrition, urinary catheter, etc), surgical procedures, as well as prolonged hospitalization are associated with CREC development [11]. (2) ECC is characterized with remarkable ability to acquire resistance determinants, leading to a rapid increase of CREC [12].

In terms of the resistance profiles, 100.0 and 77.8% of the CREC isolates in our hospital were resistant to imipenem and meropenem respectively. CREC isolates showed heterogeneity in resistance patterns to imipenem and meropenem, with some isolates resistant to both imipenem and meropenem while other isolates resistant to imipenem and susceptible to meropenem. This indicates that multiple mechanisms may participate in carbapenem resistance of CREC isolates, such as production of carbapenemase; Besides, over-expression of multidrug efflux pumps and loss of outer membrane protein were reported to be associated with resistance to meropenem and imipenem respectively. For the imipenem-resistant and meropenem-susceptible isolates, NDM-1 carbapenemase and loss of outer membrane protein may be the reasons explaining this resistant phenotype, which needs to be verified in the future.

Moreover, CREC isolates showed medium sensitivity to gentamicin, sulfamethoxazole-trimethoprim, nitrofurantoin and tobramycin, and high sensitivity to tigecycline, polymyxin B and amikacin. These results demonstrated that there were limited treatment options for CREC, making it a threat of drug resistance. Combination of antibiotics, such as meropenem, polymyxin B, tigecycline, and amikacin showed promising synergy results [13, 14]. However, optimal treatment combinations for different sequence types and resistant genotypes should be further evaluated. In addition, isolate CMU2 containing NDM-5 gene alone showed highly susceptibility to third and fourth generation cephalosporins as well as other antibiotics except meropenem and imipenem, which is unusual for carbapenemase producing organisms. Further investigation into the underlying mechanism is warranted.

Furthermore, our study revealed that the overall 30-day crude mortality of CREC infection was 17.4%. Previous meta-analysis showed that pooled crude mortality of carbapenem resistant *Klebsiella pneumoniae* was 42.1% [15]. Other studies reported the 30-day mortality of carbapenem-resistant *Pseudomonas aeruginosa* was 36.6% [16], whereas the mortality of carbapenem-resistant *A. baumannii* ranged from 16 to 76% [17], which were higher than the crude mortality of CREC in our hospital. Besides, all four patients who died were accompanied with other serious infections. Taken together, these indicated that CREC may be a low-virulence pathogen which deserves further validation.

Molecular epidemiology analyses were undertaken for the 18 CREC strains from our hospital. MLST analysis revealed diverse sequence types with ST93 as the predominant type followed by ST171 and ST145. ST93 was frequently reported in China, such as Hangzhou, Nanjing, Jiamusi, etc. [18–20], whereas ST171 was commonly reported the U.S.A. and Japan [21, 22]. The diversification of the sequence types of CREC in our hospital is consistent with previous studies which also showed genetic heterogeneity [10]. For the resistance genes, NDM-1 and NDM-5 were the predominant carbapenemase-encoding genes, and TEM-1 was the most common β-lactamase gene.

CREC is an emerging multi-drug resistant pathogen, which are associated with the risk of spreading to the communities. Therefore, it is imperative to take effective infection prevention and control practices to confront this threat [21]. However, the wide genotypic diversity of CREC isolates may indicate that CREC has strong ability to acquire drug resistance genes, thereby increasing the difficulty in infection prevention and control. Above all, screening of carbapenem resistant pathogens should be conducted, especially in patients with high risks. Moreover, other infection and control measures, including rational use of antibiotics, environment cleaning, faecal and medical waste management, hand hygiene, staff education, etc. should be implemented to curb the global spread of CREC [23].

The current study has some limitations. Firstly, the number of isolates is relatively small because CREC were infrequent despite the increasing detection rate in our hospital. Secondly, this was a retrospective study in a single center, and 11 out of 29 CREC isolates were not cryopreserved, which may lead to bias. Thirdly, although the mortality of CREC is low compared with other carbapenem resistant pathogens, which indicates its low virulence, further virulence-based experiments should be performed.

In conclusion, a rapid increase of CREC was observed during 2010–2019 in our hospital, which were with limited treatment options. Molecular epidemiology demonstrated the diversification of CREC, with ST93 as the predominant sequence type and NDM as the main carbapenemase-encoding gene. Intensive surveillance and effective measures should be undertaken to reduce the spread and transmission of CREC in the hospitals.

## Supplementary Information

**Additional file 1: Table S1**. Primers for polymerase chain reactions in this present study. **Table S2**. MLST analysis of 18 CREC isolates.

## Data Availability

All data generated or analyzed during this study were included in this article and the supplementary information files. The phylogenetic datasets analyzed during the current study are available in the TreeBASE repository (http://purl.org/phylo/treebase/phylows/study/TB2:S28058, accession number: 28058).

## Abbreviations

CREC: Carbapenem-resistant *Enterobacter cloacae* complex
ECC: *Enterobacter cloacae* complex
CRE: Carbapenem-resistant Enterobacteriaceae
CHINET: China Antimicrobial Surveillance network
MICs: Minimal inhibitory concentrations
EUCAST: European Committee on Antimicrobial Susceptibility Testing
CLSI: Clinical and Laboratory Standards Institute
PCR: Polymerase chain reaction
MLST: multilocus sequence typing
ST: Sequence type
