# Supplementary material for: Carbapenem-resistant Enterobacter cloacae complex in a tertiary Hospital in Northeast China, 2010–2019
Source: BMC Infect Dis. 2021 Jun 26;21:611. doi: 10.1186/s12879-021-06250-0 (PMC8235818; doi:10.1186/s12879-021-06250-0)
Supplement: Supplementary file 1 — Additional file 1: Table S1. Primers for polymerase chain reactions in this present study. Table S2. MLST analysis of 18 CREC isolates. [file 12879_2021_6250_MOESM1_ESM.docx]

Table S1 Primers for polymerase chain reactions in this present study

| Primers | Sequence (5 ′ –3 ′) | References |
| --- | --- | --- |
| KPC-Fm | CGTCTAGTTCTGCTGTCTTG | Poirel et al., 2011 |
| KPC-Rm | CTTGTCATCCTTGTTAGGCG |  |
| IMP-F | GGAATAGAGTGGCTTAAYTCTC | Poirel et al., 2011 |
| IMP-R | GGTTTAAYAAAACAACCACC |  |
| VIM-F | GATGGTGTTTGGTCGCATA | Poirel et al., 2011 |
| VIM-R | CGAATGCGCAGCACCAG |  |
| OXA-F | GCGTGGTTAAGGATGAACAC | Poirel et al., 2011 |
| OXA-R | CATCAAGTTCAACCCAACCG |  |
| NDM-F | GGTTTGGCGATCTGGTTTTC | Poirel et al., 2011 |
| NDM-R | CGGAATGGCTCATCACGATC |  |
| *bla*_TEM_*_*F | TTAGACGTCAGGTGGCACTT | Biendo et al., 2008 |
| *bla*_TEM_*_*R | GGACCGGAGTTACCAATGCT |  |
| *bla*_CTX-M_*_*F | ATGTGCAGYACCAGTAARGT | Pagani et al., 2003 |
| *bla*_CTX-M_*_*R | TGGGTRAARTARGTSACCAGA |  |
| *bla*_SHV_*_*F | TCGGCCTTCACTCAAGGATG | Biendo et al., 2008 |
| *bla*_SHV__R | GGACCGGAGTTACCAATGCT |  |
| *mcr_*F | CGGTCAGTCCGTTTGTTC | Liu et al., 2016 |
| *mcr_*R | CTTGGTCGGTCTGTAGGG |  |
| dnaA-f2 | AYAACCCGCTGTTCCTBTATGGCGGCAC | Miyoshi-Akiyama, et al., 2013 |
| dnaA-r | KGCCAGCGCCATCGCCATCTGACGCGG |  |
| fusA-f2 | TCGCGTTCGTTAACAAAATGGACCGTAT | Miyoshi-Akiyama, et al., 2013 |
| fusA-r2 | TCGCCAGACGGCCCAGAGCCAGACCCAT |  |
| gyrB-f | TCGACGAAGCGCTCGCGGGTCACTGTAA | Miyoshi-Akiyama, et al., 2013 |
| gyrB-r | GCAGAACCGCCCGCGGAGTCCCCTTCCA |  |
| leuS-f2 | GATCARCTSCCGGTKATCCTGCCGGAAG | Miyoshi-Akiyama, et al., 2013 |
| leuS-r | ATAGCCGCAATTGCGGTATTGAAGGTCT |  |
| pyrG-f | AYCCBGAYGTBATTGCRCAYMAGGCGAT | Miyoshi-Akiyama, et al., 2013 |
| pyrG-r | GCRCGRATYTCVCCCTSHTCGTCCCAGC |  |
| rplB-f | GTAAACCGACATCTCCGGGTCGTCGCCA | Miyoshi-Akiyama, et al., 2013 |
| rplB-r | ACCTTTGGTCTGAACGCCCCACGGAGTT |  |
| rpoB-f | AAAAACGTATTCGTAAGGATTTTGGTAA | Miyoshi-Akiyama, et al., 2013 |
| rpoB-r2 | CCAGCAGATCCAGGCTCAGCTCCATGTT |  |
| 519F | CAGCMGCCGCGGTAATWC |  |
| 1406R | ACGGGCGGTGTGTRC |  |
| Hsp60-F | GGTAGAAGAAGGCGTGGTTGC | Hoffmann, et al., 2003 |
| Hsp60-R | ATGCATTCGGTGGTGATCATCAG |  |

Table S2 MLST analysis of 18 CREC isolates

| Sample ID | Allelic type | | | | | | | Sequence type |
| --- | --- | --- | --- | --- | --- | --- | --- | --- |
|  | **dnaA** | **fusA** | **gyrB** | **leuS** | **pyrG** | **rplB** | **rpoB** |  |
| CMU2 | 84 | 62 | 95 | 98 | 94 | 43 | 52 | ST250 |
| CMU5 | 59 | 40 | 82 | 9 | 67 | 6 | 6 | ST145 |
| CMU6 | 9 | 4 | 14 | 61 | 37 | 4 | 9 | ST93 |
| CMU8 | 52 | 21 | 20 | 44 | 45 | 4 | 6 | ST66 |
| CMU10 | 9 | 4 | 14 | 61 | 37 | 4 | 9 | ST93 |
| CMU11 | 9 | 4 | 14 | 61 | 37 | 4 | 9 | ST93 |
| CMU12 | 9 | 4 | 14 | 61 | 37 | 4 | 9 | ST93 |
| CMU13 | 13 | 2 | 45 | 27 | 56 | 2 | 14 | ST13 |
| CMU14 | 49 | 21 | 19 | 44 | 45 | 12 | 32 | ST171 |
| CMU15 | 59 | 40 | 82 | 9 | 67 | 6 | 6 | ST145 |
| CMU18 | 53 | 35 | 20 | 44 | 45 | 4 | 6 | ST114 |
| CMU19 | 95 | 56 | 112 | 116 | 104 | 4 | 63 | ST528 |
| CMU23 | 49 | 21 | 19 | 44 | 45 | 12 | 32 | ST171 |
| CMU25 | 9 | 4 | 344 | 61 | 89 | 4 | 9 | ST1120 |
| CMU26 | 9 | 4 | 14 | 61 | 37 | 4 | 9 | ST93 |
| CMU27 | 49 | 21 | 19 | 44 | 45 | 12 | 32 | ST171 |
| CMU28 | 9 | 4 | 14 | 61 | 37 | 4 | 9 | ST93 |
| CMU29 | 9 | 4 | 14 | 61 | 37 | 4 | 9 | ST93 |
